# Supplementary material for: Stiffness-switchable DNA-based constitutional dynamic network hydrogels for self-healing and matrix-guided controlled chemical processes
Source: Nat Commun. 2019 Oct 21;10:4774. doi: 10.1038/s41467-019-12697-2 (PMC6803638; doi:10.1038/s41467-019-12697-2)
Supplement: Supplementary file 1 — Supplementary Information [file 41467_2019_12697_MOESM1_ESM.pdf]

Supplementary Information

Stiffness-Switchable DNA-Based Constitutional Dynamic Network Hydrogels for Self-Healing and Matrix-Guided Controlled Chemical Processes

Yue et al.

Table of Content

Supplementary Table 1. Effective Young's moduli corresponding to the CDN hydrogels……..…...**4**

Supplementary Fig. 1. Reversible stiffness properties of the CDN hydrogels………………..….….…**4**

Supplementary Fig. 2. Rheological properties of the CDN hydrogels……………………………...….**5**

Supplementary Fig. 3. Rheometric characterization of the concentration effect of E_2 on the transition of CDN hydrogel X………………………………………………………….………..…...….**6**

Supplementary Fig. 4. Rheometric characterization of the time-dependent transitions of the CDN hydrogels………………………………………………………………………………....….**6**

Determination of the concentrations of the constituents in the CDN hydrogels (including Supplementary Figs. 5−12)………………………………...…………………………......…..**7**

Supplementary Fig. 13. Reversible and switchable transition between CDN X and CDN Y…....….**13**

Supplementary Fig. 14. Reversible and switchable transition between CDN X and CDN Z……....**14**

Further support that the compositions of the model CDNs represent the compositions of the CDN hydrogels (including Supplementary Figs. 15 and 16)………………………………………..**14**

Supplementary Fig. 17. Visual imaging of the transition of CDN hydrogel Z back to X……….....…**16**

Supplementary Fig. 18. Visual imaging of the transitions of CDN hydrogels (X → Y → Z)…………………………………………………………………………………..………..**17**

Supplementary Fig. 19. Rheometric characterization of the self-healing ability of the CDN hydrogel…………………………………………………………………………………………..**17**

Supplementary Fig. 20. Self-healing of CDN hydrogels X and Z………………………….……..…..**18**

Supplementary Fig. 21. Determination of the concentration of glucose oxidase (GOx) loading in the CDN hydrogel X matrix……………………………………………………………………...…...**18**

Supplementary Fig. 22. Determination of the concentration of horseradish peroxidase (HRP) loading in the CDN hydrogel X matrix……………………...…**19**

Supplementary Fig. 23. Catalytic activities of the bi-enzyme cascade reaction corresponding to the healed or non-healed GOx/HRP-loaded hydrogel matrices……………………………………......….**20**

Supplementary Fig. 24. Control experiment of the leakage of GOx/HRP from the healed GOx/HRP-loaded hydrogel matrix..**20**

Supplementary Fig. 25. Catalytic activities of the bi-enzyme cascade reaction in a single GOx/HRP-loaded hydrogel matrix……………………………………………………...………......…..**21**

Supplementary Fig. 26. Triggered release of doxorubicin by controlling the crosslinking of CDN hydrogel Y……………………………………………………………………..........…..**21**

Insight into the loading of doxorubicin in hydrogel X and its release profile (including Supplementary Figs. 27 and 28)…………………………...………………………..…......…..**22**

Supplementary Table 1 | Effective Young's moduli corresponding to the CDN hydrogels: (i) Hydrogel X; (ii) Hydrogel Y; (iii) Hydrogel Z; (iv) The regenerated hydrogel X from hydrogel Y; (v) The regenerated hydrogel X from hydrogel Z. The data were given as the mean of about 100 indentations and its standard error.

System	Y_{eff} (kPa)
i	13.18 ± 0.29
ii	23.45 ± 0.43
iii	3.75 ± 0.03
iv	11.05 ± 0.03
v	11.14 ± 0.06

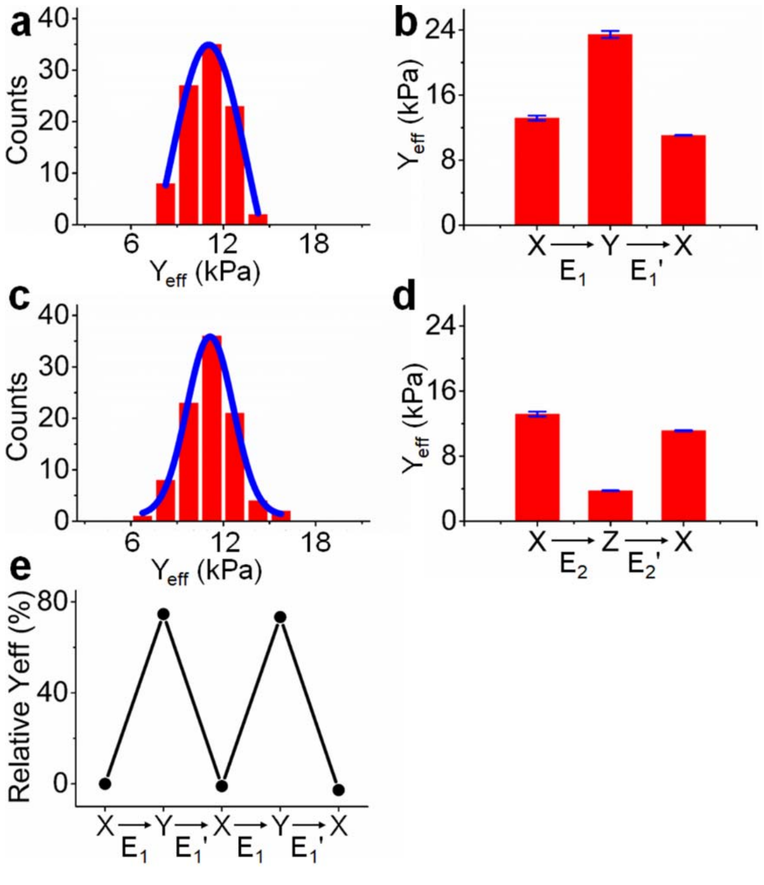

Supplementary Fig. 1 | Reversible stiffness properties of the CDN hydrogels. a, Histograms and corresponding Gaussian fit of effective Young's moduli associated with the regenerated CDN hydrogel X from hydrogel Y. **b**, Young's moduli, in the form of a "bar" presentation, corresponding to hydrogel X and hydrogel Y, and the regenerated hydrogel X from hydrogel Y (given as the mean

of about 100 indentations and its standard error). **c**, Histograms and corresponding Gaussian fit of effective Young's moduli associated with the regenerated CDN hydrogel X from hydrogel Z. **d**, Young's moduli, in the form of a "bar" presentation, corresponding to hydrogel X and hydrogel Z, and the regenerated hydrogel X from hydrogel Z (given as the mean of about 100 indentations and its standard error). It should be noted that the one-cycle, reversible transition between the hydrogel states upon the addition of the effectors E_1/E_1' or E_2/E_2' results in a ca. 10% decrease in the respective Young's moduli, as compared to the initial value of hydrogel X. This is due to the dilution of the hydrogel solution upon the addition of the respective effectors (6.3 μL of 4 mM for each trigger per 100 μL of hydrogel). By concentrating the added effectors (2.5 μL of 10 mM for each trigger per 100 μL of hydrogel), this loss in the stiffness was diminished. **e**, Young's moduli changes upon two-cycle transition between hydrogels X and Y by using concentrated effectors E_1/E_1'. Relative $Y_{eff} = (Y_{eff} - Y_{eff(X)})/Y_{eff(X)} \times 100\%$, where $Y_{eff(X)}$ represents Young's modulus of hydrogel X without any triggers. One may realize the Young's moduli of the restored hydrogel X decreased by ca. 3% after completing two cycles. Thus, one may conclude that the dilution of the hydrogel by the added effectors could affect the long-term, multi-cycle, switchable properties of the CDN hydrogel.

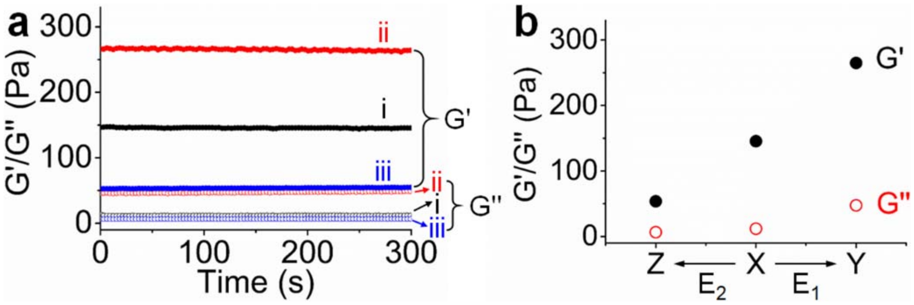

Supplementary Fig. 2 | Rheological properties of the CDN hydrogels. a, G′ and G″ values of (i) hydrogel X; (ii) hydrogel Y; (iii) hydrogel Z. **b,** G′ and G″ values of the different hydrogels derived from the data shown in **a**.

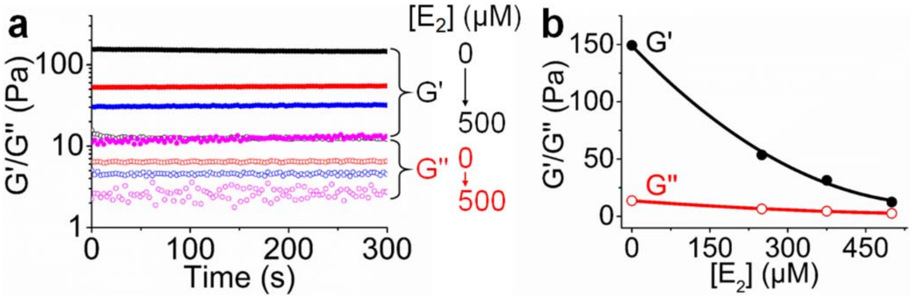

Supplementary Fig. 3 | Rheometric characterization of the concentration effect of E_2 on the transition of CDN hydrogel X. a, G′ and G″ values of hydrogel X upon the addition of E_2 at different concentrations: 0 μM, 250 μM, 375 μM and 500 μM. **b,** G′ and G″ value changes of the resulting systems as a function of the concentration of E_2, derived from the data shown in **a**.

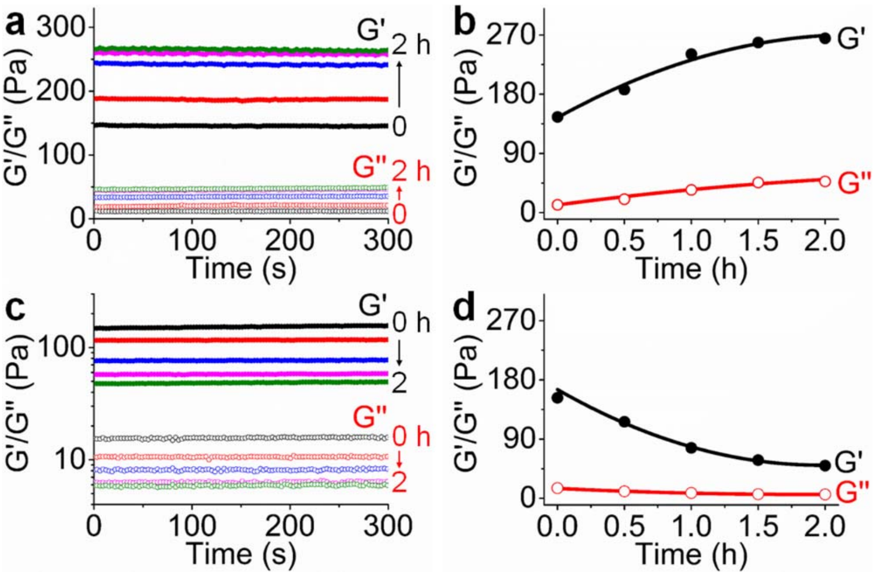

Supplementary Fig. 4 | Rheometric characterization of the time-dependent transitions of the CDN hydrogels. a, G′ and G″ values corresponding to the E_1-triggered transition of hydrogel X to hydrogel Y at different time intervals. **b,** The time-dependent G′ and G″ changes of the E_1-triggered transition of hydrogel X to hydrogel Y, derived from the data shown in **a**. **c,** G′ and G″ values corresponding to the E_2-triggered transition of hydrogel X to hydrogel Z at different time intervals. **d,** The time-dependent G′ and G″ value changes of the E_2-triggered transition of hydrogel X to hydrogel Z, derived from the data shown in **c**.

Determination of the concentrations of the constituents in the CDN hydrogels

In order to evaluate the contents of the constituents in the different hydrogel configurations, we designed a model CDN system, X_m, where the toehold sequences leading to the hydrogelation of CDN X are eliminated by replacing L_1 and L_2 with L_{1m} and L_{2m}, respectively (Supplementary Fig. 5). Under these conditions, CDN X_m is still reconfigured to CDN Y_m or Z_m with the same triggers used to reequilibrate the respective hydrogels. The method to evaluate the contents of the constituents in CDNs X_m, Y_m and Z_m involved the labeling of the constituents and the application of the following process.

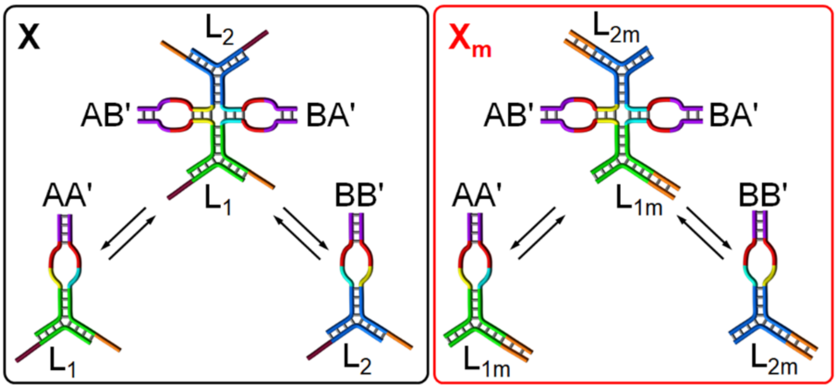

Supplementary Fig. 5 | Schematic presentation of the compositions of CDN X and the model CDN X_m. The toehold sequences leading to the hydrogelation of CDN X are eliminated in the model CDN X_m by replacing L_1 and L_2 with L_{1m} and L_{2m}, respectively. It should be noted that the strands L_1, L_2, L_{1m} and L_{2m} do not participate in the equilibria of the CDNs.

The procedure to quantify the concentrations of the constituents in CDN hydrogels X, Y and Z includes the application of two sets of fluorophore-labeled model CDNs (X_m, Y_m and Z_m). In one set, Supplementary Fig. 6, constituent AA' is labeled with Cy3 (λ_{ex} = 545 nm; λ_{em} = 565 nm), constituent BB' is labeled with Cy5 (λ_{ex} = 620 nm; λ_{em} = 665 nm) and constituent AB' is labeled with a Cy3/Cy5 fluorophore pair that leads to a FRET signal. Recording the fluorescence spectra of these CDNs yields the following fluorescence intensity relations:

a) The fluorescence observed upon exciting the CDNs at λ_{ex} = 545 nm and monitoring the fluorescence intensities at λ_{em} = 565 nm ($F_{545\text{-}565}$) is generated by constituents AA' and AB'.

$$F_{545\text{-}565} = F_{AA'\,(Cy3)\,545\text{-}565} + F_{AB'\,(Cy3)\,545\text{-}565} \qquad \text{Supplementary eq. 1}$$

b) The fluorescence observed upon exciting the CDNs at λ_{ex} = 620 nm and monitoring the fluorescence intensities at λ_{em} = 665 nm ($F_{620\text{-}665}$) is generated by constituents BB' and AB'.

$$F_{620\text{-}665} = F_{BB'\,(Cy5)\,620\text{-}665} + F_{AB'\,(Cy5)\,620\text{-}665} \qquad \text{Supplementary eq. 2}$$

c) The fluorescence observed upon exciting the CDNs at λ_{ex} = 545 nm and monitoring the fluorescence intensities at λ_{em} = 665 nm ($F_{545\text{-}665}$) is generated by BB', AB' and AA' (residual emission at λ_{em} = 665 nm of Cy3).

$$F_{545\text{-}665} = F_{AA'\,(Cy3)\,545\text{-}665} + F_{BB'\,(Cy5)\,545\text{-}665} + F_{AB'\,(Cy3\text{-}Cy5)\,545\text{-}665} \qquad \text{Supplementary eq. 3}$$

With the help of appropriate fluorescence calibration curves for the intact constituents AA', BB' and AB' labeled with fluorophores (Supplementary Figs. 7 and 8) and the set of Supplementary equations 1–3, the concentrations of the three constituents AA', BB' and AB' can be determined by measuring the fluorescence spectra of the respective CDNs.

Supplementary Fig. 9a shows the fluorescence spectra generated from the fluorophore-labeled model CDNs X_m, Y_m and Z_m at λ_{ex} = 545 nm. The spectra show that the fluorescence intensity at λ_{em} = 565 nm is lower for CDN Y_m as compared to that of CDN X_m, while the fluorescence intensity of CDN Z_m is higher than that of CDN X_m. In addition, the fluorescence intensity at λ_{em} = 665 nm is lowered for CDN Z_m as compared to that of CDN X_m, whereas the fluorescence of CDN Y_m is intensified as compared to that of CDN X_m. Supplementary Fig. 9b shows the fluorescence spectra generated from the model CDNs X_m, Y_m and Z_m at λ_{ex} = 620 nm. The spectra show that the fluorescence intensity at λ_{em} = 665 nm is lower for CDN Y_m as compared to that of CDN X_m, while the fluorescence intensity of CDN Z_m is higher than that of CDN X_m.

The concentrations of the constituents AA', BB', AB' in the different CDNs were derived from the results shown in Supplementary Fig. 9a,b, using the appropriate calibration curves (Supplementary Figs. 7 and 8) and the set of Supplementary equations 1–3. The calculated concentrations are presented in the form of a bar diagram (Supplementary Fig. 9c) and summarized in Table 1.

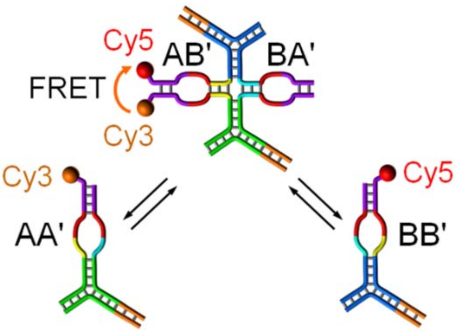

Supplementary Fig. 6 | First schematic set of the Cy3/Cy5-labeled model CDN X_m for determination of the compositions of the CDN hydrogels. Constituent AA' is labeled with Cy3, constituent BB' is labeled with Cy5 and constituent AB' is labeled with a Cy3/Cy5 fluorophore pair that leads to a FRET signal. CDN X_m is reconfigured to CDN Y_m or Z_m using trigger E_1 or E_2, respectively.

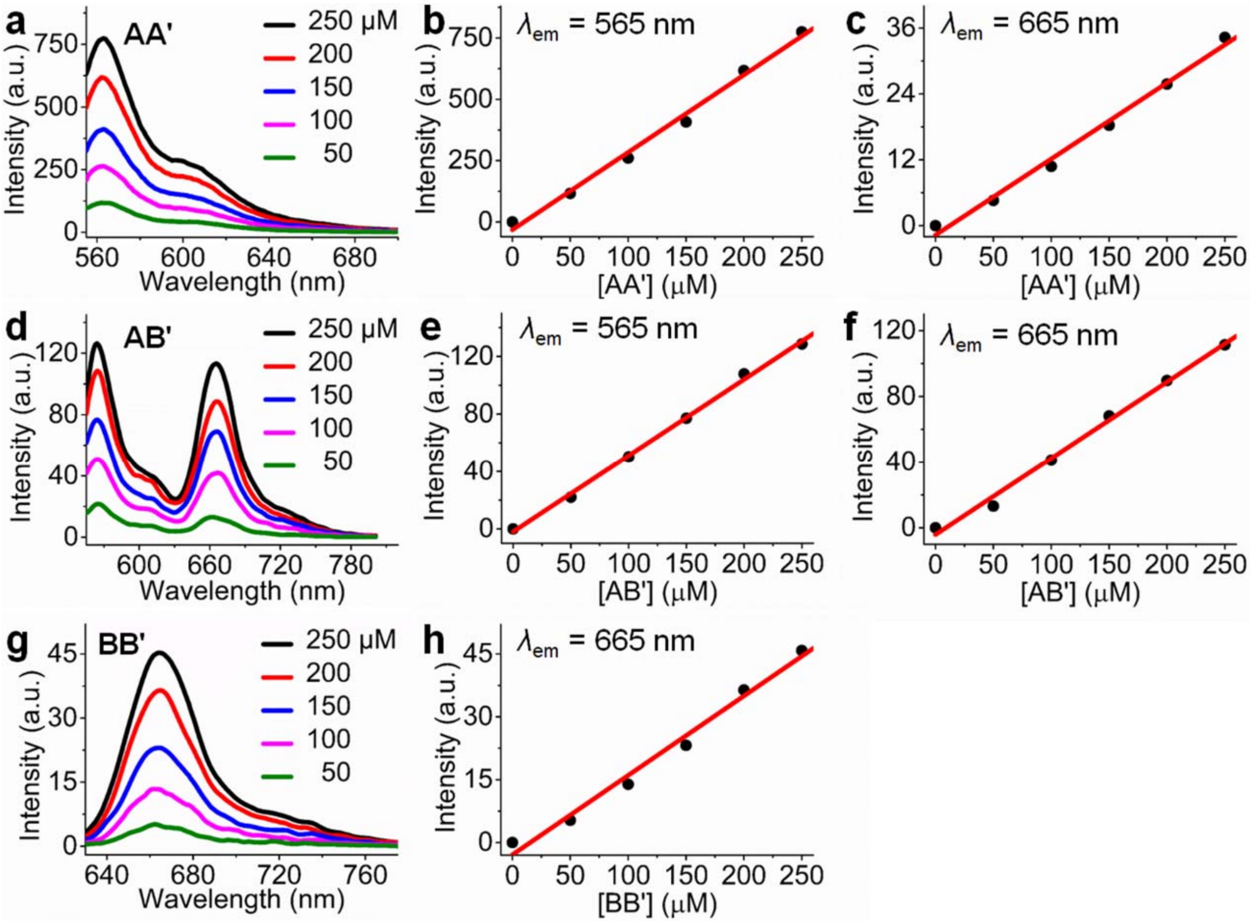

Supplementary Fig. 7 | Calibration curves (λ_{ex} = 545 nm). a, Fluorescence spectra (λ_{ex} = 545 nm) of Cy3-labeled constituent AA' at different concentrations. Corresponding calibration curves of the fluorescence intentsities: **b**, at λ_{em} = 565 nm and **c**, at λ_{em} = 665 nm, of Cy3-labeled constituent AA'

as a function of its concentration, derived from the data shown in **a**. **d**, Fluorescence spectra (λ_{ex} = 545 nm) of Cy3/Cy5-labeled constituent AB' at different concentrations. Corresponding calibration curves of the fluorescence intensities: **e**, at λ_{em} = 565 nm and **f**, at λ_{em} = 665 nm, of Cy3/Cy5-labeled constituent AB' as a function of its concentration, derived from the data shown in **d**. **g**, Fluorescence spectra (λ_{ex} = 545 nm) of Cy5-labeled constituent BB' at different concentrations. **h**, Corresponding calibration curve of the fluorescence intensity at λ_{em} = 665 nm of Cy5-labeled constituent BB' as a function of its concentration, derived from the data shown in **g**. Note that all the samples were measured upon a 1 : 250 dilution.

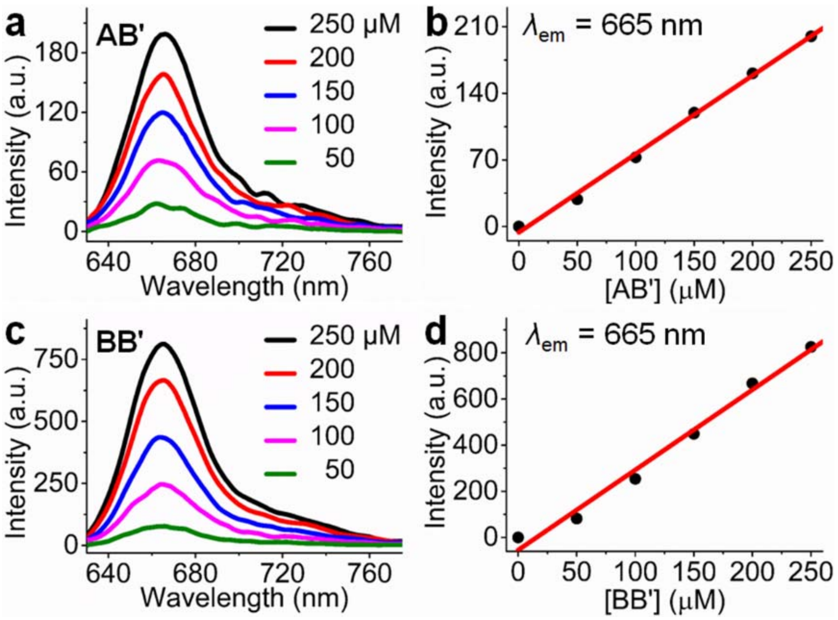

Supplementary Fig. 8 | Calibration curves (λ_{ex} = 620 nm). a, Fluorescence spectra (λ_{ex} = 620 nm) of Cy3/Cy5-labeled constituent AB' at different concentrations. **b**, Corresponding calibration curve of the fluorescence intenstity at λ_{em} = 665 nm of Cy3/Cy5-labeled constituent AB' as a function of its concentration, derived from the data shown in **a**. **c**, Fluorescence spectra (λ_{ex} = 620 nm) of Cy5-labeled constituent BB' at different concentrations. **d**, Corresponding calibration curve of the fluorescence intenstity at λ_{em} = 665 nm of Cy5-labeled constituent BB' as a function of its concentration, derived from the data shown in **c**. Note that all the samples were measured upon a 1 : 250 dilution.

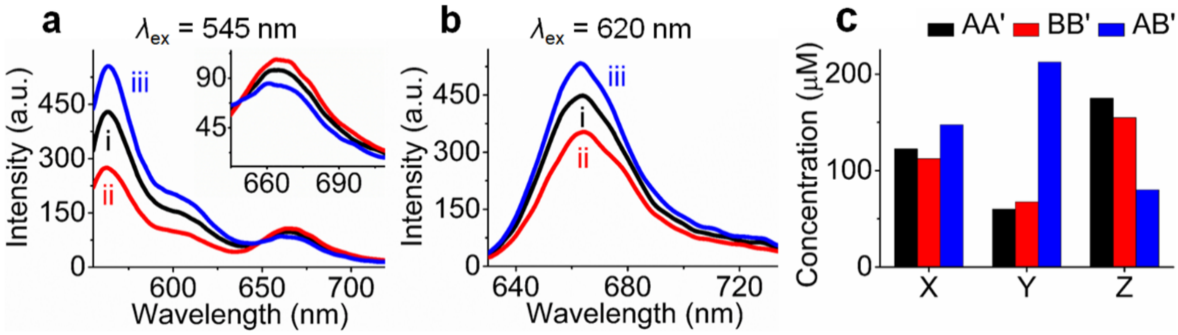

Supplementary Fig. 9 | Determination of the concentrations of the constituents AA', BB' and AB' in the different CDNs. Fluorescence spectra of the fluorophore labels: **a**, at λ_{ex} = 545 nm and **b**, at λ_{ex} = 620 nm, in CDN X_m (curves i), CDN Y_m (curves ii) and CDN Z_m (curves iii). **c**, The concentrations of the constituents AA', BB' and AB' in CDNs X, Y and Z, derived from the data shown in **a** and **b**.

In the other set, Supplementary Fig. 10, constituents AA' and BA' are labeled with FAM (λ_{ex} = 450 nm; λ_{em} = 520 nm). Probing the fluorescence features of these CDNs yields the following fluorescence intensity relations:

The fluorescence observed upon exciting the CDNs at λ_{ex} = 450 nm and monitoring the fluorescence intensities at λ_{em} = 520 nm ($F_{450\text{-}520}$) is generated by constituents AA' and BA'.

$$F_{450\text{-}520} = F_{AA'\,(FAM)\,450\text{-}520} + F_{BA'\,(FAM)\,450\text{-}520} \qquad \text{Supplementary eq. 4}$$

With the help of appropriate fluorescence calibration curves for the intact separated constituents AA' and BA' labeled with FAM (Supplementary Fig. 11), the concentrations of AA' obtained from the previous experiments and Supplementary equation 4, the concentrations of BA' in different CDNs can be determined.

Supplementary Fig. 12a shows the fluorescence spectra of fluorophore-labeled model CDNs X_m, Y_m and Z_m at λ_{ex} = 450 nm. The spectra show that the fluorescence intensities at λ_{em} = 520 nm for all the CDNs are the same. The concentrations of BA' in the different CDNs were derived from the results shown in Supplementary Fig. 12a, using the appropriate calibration curves (Supplementary Fig. 11), Supplementary equation 4 and the concentrations of AA' obtained from the previous experiments. The calculated concentrations of BA' in different CDNs are presented in the form of a bar diagram (Supplementary Fig. 12b) and summarized in Table 1.

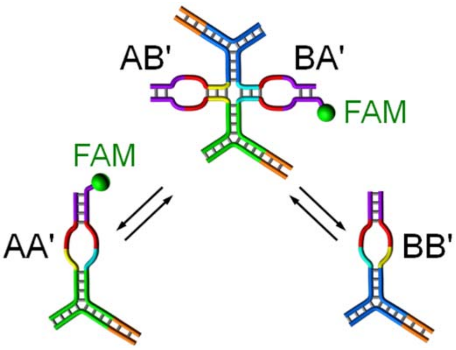

Supplementary Fig. 10 | Second schematic set of the FAM-labeled model CDN X_m for determination of the compositions of the CDN hydrogels. Constituents AA' and BA' are labeled with FAM. CDN X_m is reconfigured to CDN Y_m or Z_m using trigger E_1 or E_2, respectively.

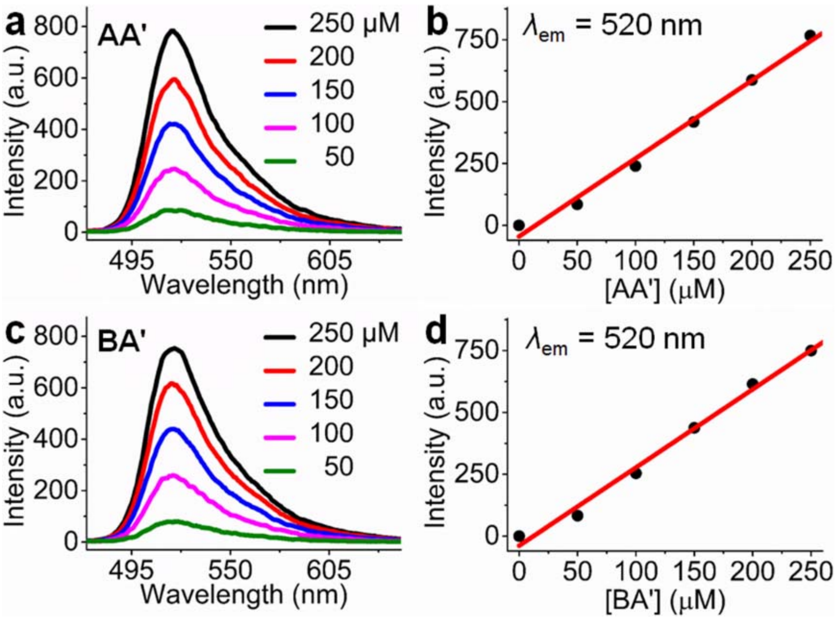

Supplementary Fig. 11 | Calibration curves (λ_{ex} = 450 nm). a, Fluorescence spectra (λ_{ex} = 450 nm) of FAM-labeled constituent AA' at different concentrations. **b**, Corresponding calibration curve of the fluorescence intensity at λ_{em} = 520 nm of FAM-labeled AA' as a function of its concentration, derived from the data shown in **a**. **c**, Fluorescence spectra (λ_{ex} = 450 nm) of FAM-labeled constituent BA' at different concentrations. **d**, Corresponding calibration curve of the fluorescence intensity at λ_{em} = 520 nm of FAM-labeled BA' as a function of its concentration, derived from the data shown in **c**. Note that all the samples were measured upon a 1 : 250 dilution.

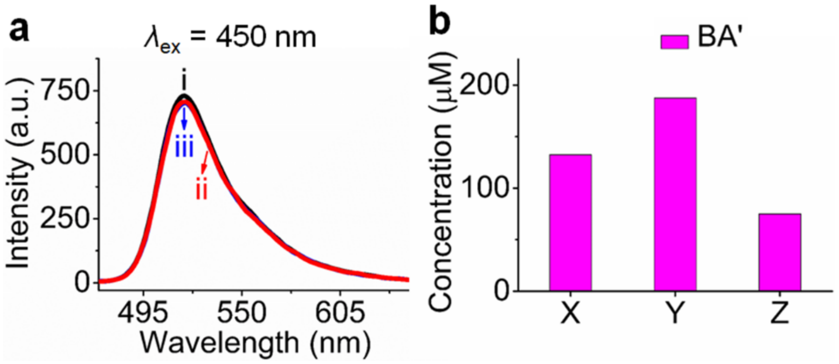

Supplementary Fig. 12 | Determination of the concentrations of constituent BA' in the different CDNs. a, Fluorescence spectra of the FAM label (λ_{ex} = 450 nm) in CDN X_m (curve i), CDN Y_m (curve ii) and CDN Z_m (curve iii). **b**, The concentrations of constituent BA' in CDNs X, Y and Z, derived from the data shown in **a**.

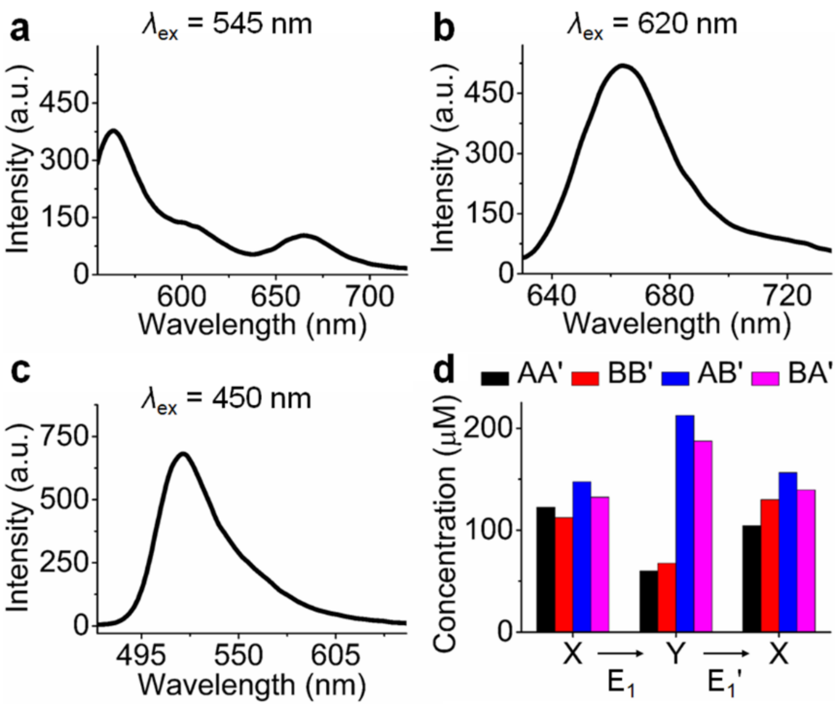

Supplementary Fig. 13 | Reversible and switchable transition between CDN X and CDN Y. Fluorescence spectra of the fluorophore labels in the regenrated model CDN X_m from CDN Y_m excited at different wavelengths: **a**, λ_{ex} = 545 nm; **b**, λ_{ex} = 620 nm and **c**, λ_{ex} = 450 nm. **d**, The concentrations of the constituents AA', BB', AB' and BA' in CDNs X and Y, and the regenerated CDN X from CDN Y.

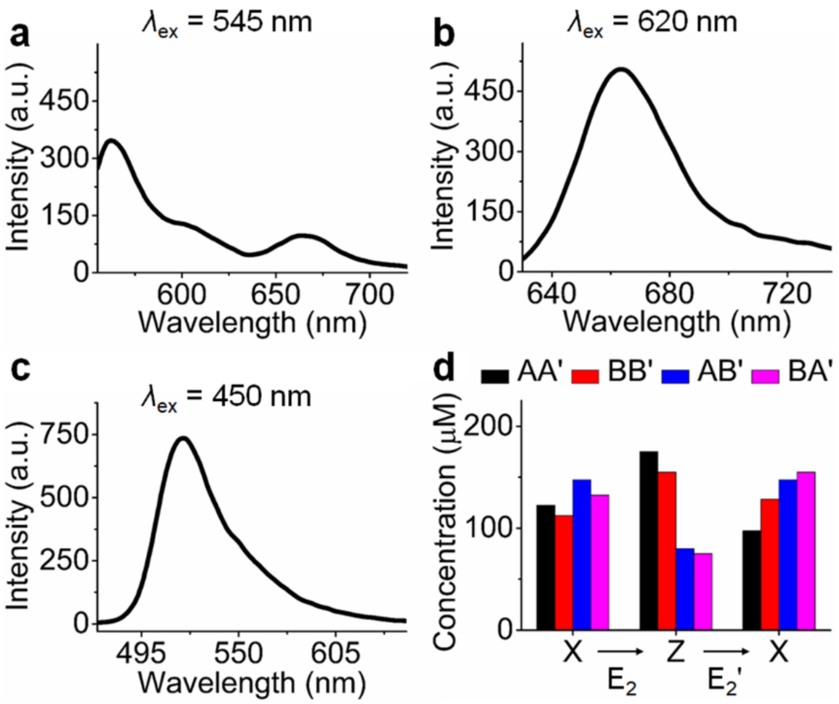

Supplementary Fig. 14 | Reversible and switchable transition between CDN X and CDN Z. Fluorescence spectra of the fluorophore labels in the regenrated model CDN X_m from CDN Z_m excited at different wavelengths: **a**, λ_{ex} = 545 nm; **b**, λ_{ex} = 620 nm and **c**, λ_{ex} = 450 nm. **d**, The concentrations of the constituents AA', BB', AB' and BA' in CDNs X and Z, and the regenerated CDN X from CDN Z.

Further support that the compositions of the model CDNs represent the compositions of the CDN hydrogels

One might argue that the evaluation of the contents of the constituents in the hydrogel systems by the model CDN analogs (X_m, Y_m and Z_m) in solution is inaccurate since the model systems include different blocker units (L_{1m} and L_{2m}) that might affect the contents of the constituents in the equilibrated mixtures. To support the use of the model CDNs as a means to evaluate the contents of the constituents in the respective hydrogels, we made use of the fact that hydrogel Z exhibits a quasi-liquid state that allows the characterization of the fluorescence properties. Accordingly, we labeled CDN hydrogel Z with the fluorophore units that were used in the labeling of model CDN Z_m, as shown in Supplementary Fig. 15. We applied the procedures outlined above to monitor the fluorescence features of the fluorophore labels in hydrogel Z. Supplementary Fig. 16a–c displays the fluorescence spectra of the labels associated with CDN hydrogel Z (curves i) and model CDN Z_m

(curves ii). One may realize that the fluorescence spectra for all labels in these two systems overlap. Thus, the contents of the constituents in CDN hydrogel Z and model CDN Z_m are identical, as presented in Supplementary Fig. 16d. These results support the use of the model CDN system to evaluate the compositions of the different hydrogels.

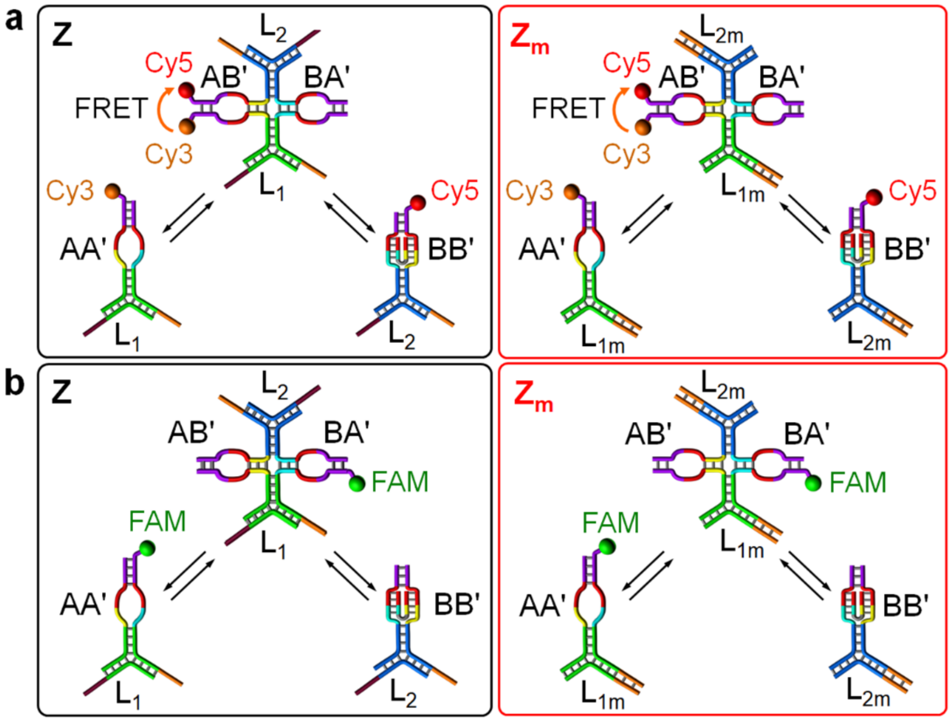

Supplementary Fig. 15 | Schematic fluorophore-labeled CDN hydrogel Z and fluorophore-labeled model CDN Z_m: a, The first set: Constituent AA' is labeled with Cy3, BB' is labeled with Cy5, and AB' is labeled with a Cy3/Cy5 pair that leads to a FRET signal. **b**, The second set: Constituents AA' and BA' are labeled with FAM.

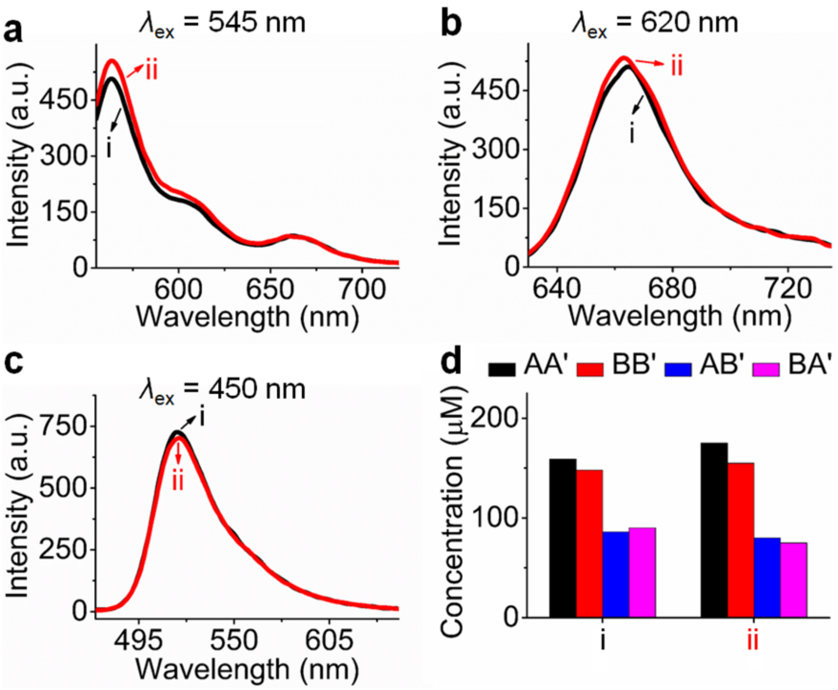

Supplementary Fig. 16 | Determination of the concentrations of the constituents in CDN hydrogel Z and model CDN Z_m. Fluorescence spectra of the fluorophore labels in CDN hydrogel Z (curves i) and model CDN Z_m (curves ii) excited at different wavelengths: **a**, λ_{ex} = 545 nm; **b**, λ_{ex} = 620 nm and **c**, λ_{ex} = 450 nm. **d**, The concentrations of the constituents AA', BB', AB' and BA' in CDN hydrogel Z (i) and model CDN Z_m (ii) evaluated from the results shown in **a**, **b** and **c**.

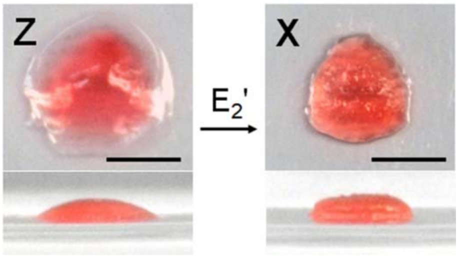

Supplementary Fig. 17 | Visual imaging of the transition of CDN hydrogel Z back to X. The E_2'-induced transition of the low-stiffness hydrogel Z back to medium-stiffness hydrogel X. Scale bars in all images correspond to 0.5 cm.

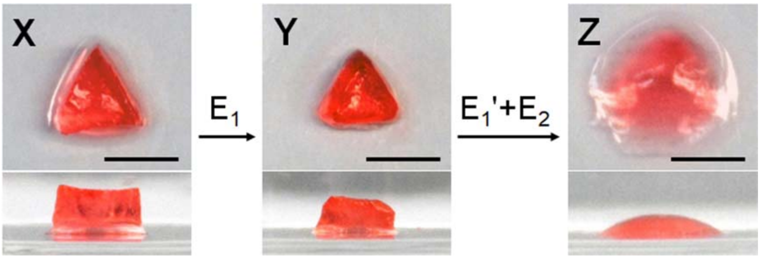

Supplementary Fig. 18 | Visual imaging of the transitions of CDN hydrogels (X → Y → Z). The E_1-induced transition of the medium-stiffness hydrogel X to the high-stiffness hydrogel Y, subsequently, transition to the low-stiffness hydrogel Z in the presence of E_1' and E_2. Scale bars in all images correspond to 0.5 cm. In this experiment, trigger E_1 (100 μL of 0.8 mM in HEPES buffer) was used to induce the transition of a triangle-shaped CDN hydrogel X (100 μL) to hydrogel Y, then triggers E_1' and E_2 (100 μL HEPES solution including 0.8 mM E_1' and 0.8 mM E_2) were applied simultaneously to stimulate the transition of the resulting hydrogel Y to hydrogel Z.

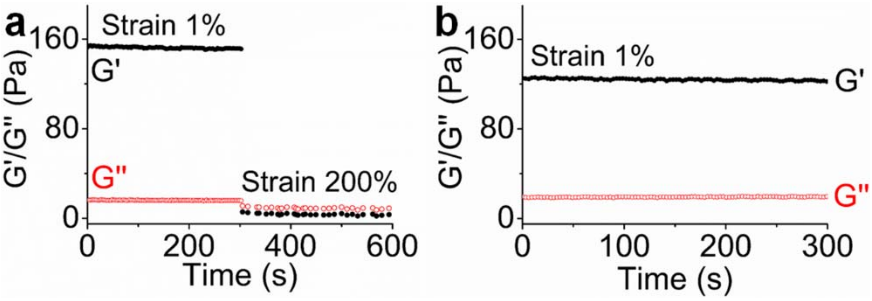

Supplementary Fig. 19 | Rheometric characterization of the self-healing ability of the CDN hydrogel. a, G′ and G″ values of hydrogel X at strains of 1% and 200%. At a strain of 200%, hydrogel X was disrupted. **b,** G′ and G″ values of the healed hydrogel X at a strain of 1%, upon subjecting the disrupted matrix to E_1 for 2 hours to heal and then to E_1' to recover state X.

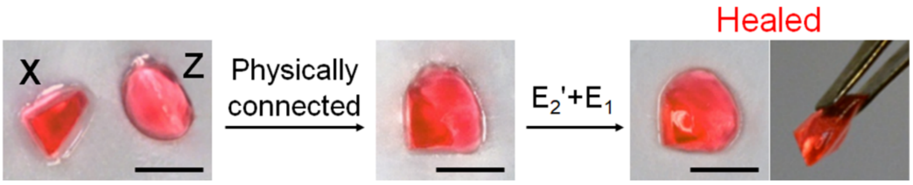

Supplementary Fig. 20 | Self-healing of CDN hydrogels X and Z. The physically interconnected hydrogels X and Z were subjected to triggers E_2' and E_1 to yield the healed hydrogel in state Y. Scale bars in all images correspond to 0.5 cm. In this experiment, triggers E_2' and E_1 (100 μL HEPES solution including 0.9 mM E_2' and 0.9 mM E_1) were applied simultaneously for healing hydrogel X (75 μL) and hydrogel Z (75 μL).

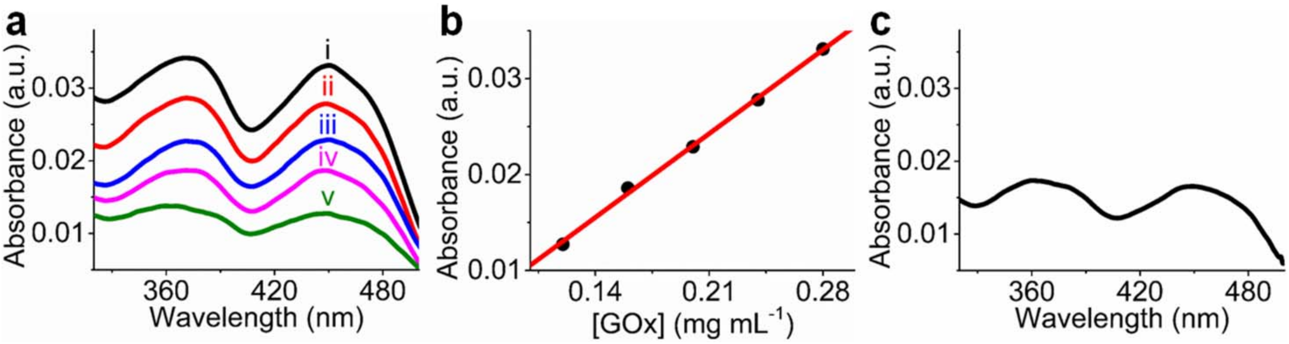

Supplementary Fig. 21 | Determination of the concentration of glucose oxidase (GOx) loading in the CDN hydrogel X matrix. a, UV-Vis spectra of GOx at different concentrations: i, 0.12 mg mL^{-1}; ii, 0.16 mg mL^{-1}; iii, 0.20 mg mL^{-1}; iv, 0.24 mg mL^{-1} and v, 0.28 mg mL^{-1}. **b**, Corresponding calibration curve of the absorbance at λ = 450 nm of GOx as a function of its concentration, derived from the data shown in **a**. **c**, UV-Vis spectra of the collected washing buffer solution (2 mL) during the loading process. According to the calibration curve in **b**, the concentration of GOx in the washing buffer solution is 0.15 mg mL^{-1}. Thus the concentration of GOx loading in the CDN hydrogel X cuboid (0.2 cm^3) was caculated to be 0.38 mg mL^{-1}. The cuboid was cut into four equal pieces, and one piece (0.05 cm^3, the loaded GOx is ~2.4 U) was used for self-healing and the activation of bi-enzyme cascade reaction.

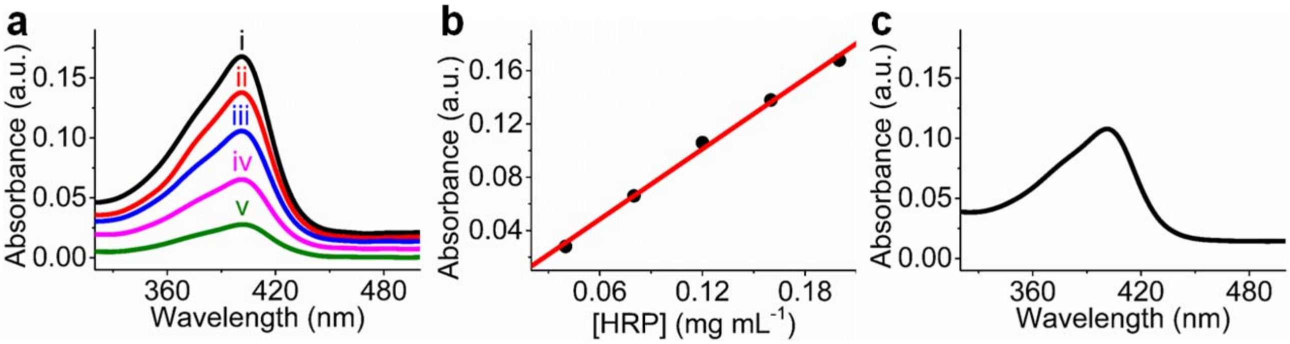

Supplementary Fig. 22 | Determination of the concentration of horseradish peroxidase (HRP) loading in the CDN hydrogel X matrix. a, UV-Vis spectra of HRP at different concentrations: i, 0.04 mg mL^{-1}; ii, 0.08 mg mL^{-1}; iii, 0.12 mg mL^{-1}; iv, 0.16 mg mL^{-1} and v, 0.20 mg mL^{-1}. **b**, Corresponding calibration curve of the absorbance at λ = 400 nm of HRP as a function of its concentration, derived from the data shown in **a**. **c**, UV-Vis spectra of the collected washing buffer solution (2 mL) during the loading process. According to the calibration curve in **b**, the concentration of HRP in the washing buffer solution is 0.13 mg mL^{-1}. Thus the concentration of HRP loading in the CDN hydrogel X cuboid (0.2 cm^3) was caculated to be 0.58 mg mL^{-1}. The cuboid was cut into four equal pieces, and one piece (0.05 cm^3, the loaded HRP is ~2.9 U) was used for self-healing and the activation of bi-enzyme cascade reaction.

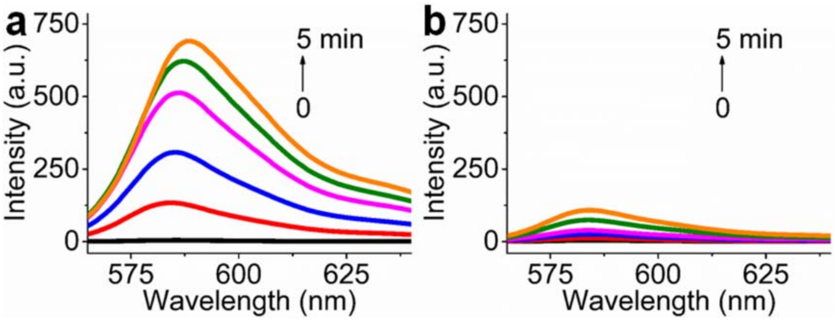

Supplementary Fig. 23 | Catalytic activities of the bi-enzyme cascade reaction corresponding to the healed or non-healed GOx/HRP-loaded hydrogel matrices. Fluorescence spectra of Resorufin upon: **a**, The operation of the GOx/HRP bi-enzyme cascade in a healed matrix composed of a GOx-loaded hydrogel matrix linked to a HRP-loaded hydrogel matrix; **b**, The operation of the bi-enzyme cascade in non-linked (separated) GOx-loaded hydrogel matrix and HRP-loaded hydrogel matrix.

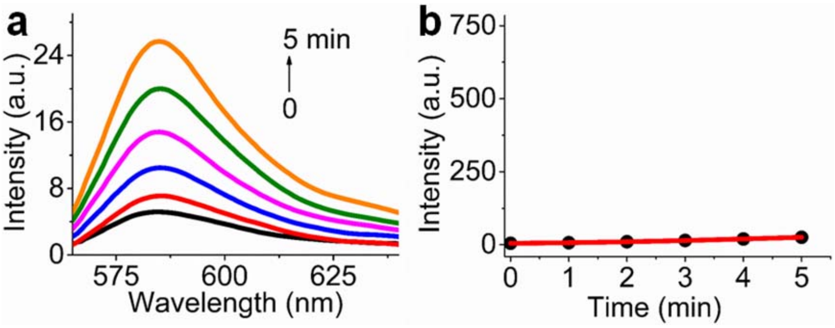

Supplementary Fig. 24 | Control experiment of the leakage of GOx/HRP from the healed hydrogel matrix. **a**, Fluorescence spectra and **b**, time-dependent fluorescence intensity changes of Resorufin upon the operation of the GOx/HRP bi-enzyme cascade in the collected HEPES buffer solution after soaking the healed GOx/HRP-loaded hydrogel matrix for one hour. The result shows negligible leakage of GOx/HRP from the healed GOx/HRP-loaded hydrogel matrix in the reaction buffer.

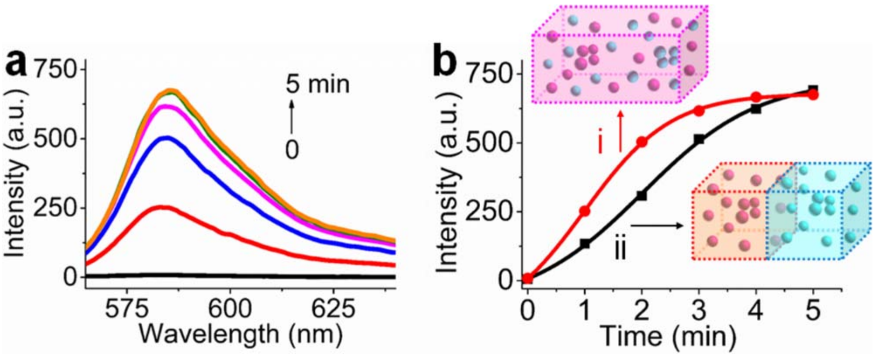

Supplementary Fig. 25 | Catalytic activities of the bi-enzyme cascade reaction in a single GOx/HRP-loaded hydrogel matrix. a, Fluorescence spectra of Resorufin upon the operation of the GOx/HRP bi-enzyme cascade combined in a single GOx/HRP-loaded hydrogel matrix at different time intervals; **b**, Time-dependent fluorescence changes generated upon: (i) The operation of the GOx/HRP bi-enzyme cascade in a single GOx/HRP-loaded hydrogel matrix; (ii) The operation of the bi-enzyme cascade in a healed matrix composed of a GOx-loaded hydrogel matrix linked to a HRP-loaded hydrogel matrix.

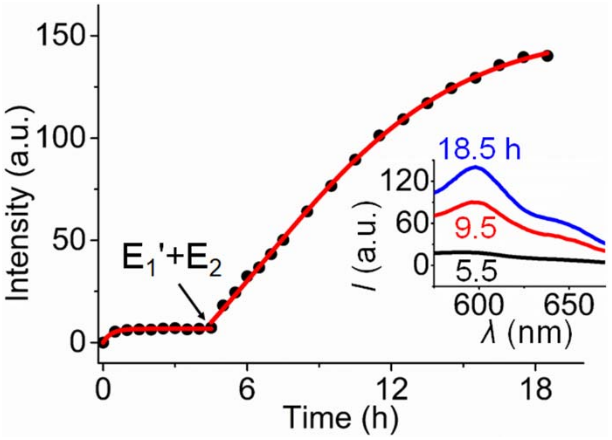

Supplementary Fig. 26 | Triggered release of doxorubicin by controlling the crosslinking of CDN hydrogel Y. Time-dependent release profile of doxorubicin from the high-stiffness hydrogel Y (200 μL). After 4.5 hours, triggers E_1' (20 μL of 4 mM) and E_2 (20 μL of 4 mM) were added to stimulate the transition of hydrogel Y to Z, to switch on the release process.

<u>Insight into the loading of doxorubicin in hydrogel X and its release profile</u>

a) The integration of doxorubicin in hydrogel X could be followed by confocal fluorescence microscopy, Supplementary Fig. 27 panel I, which shows the characteristic green fluorescence of doxorubicin.

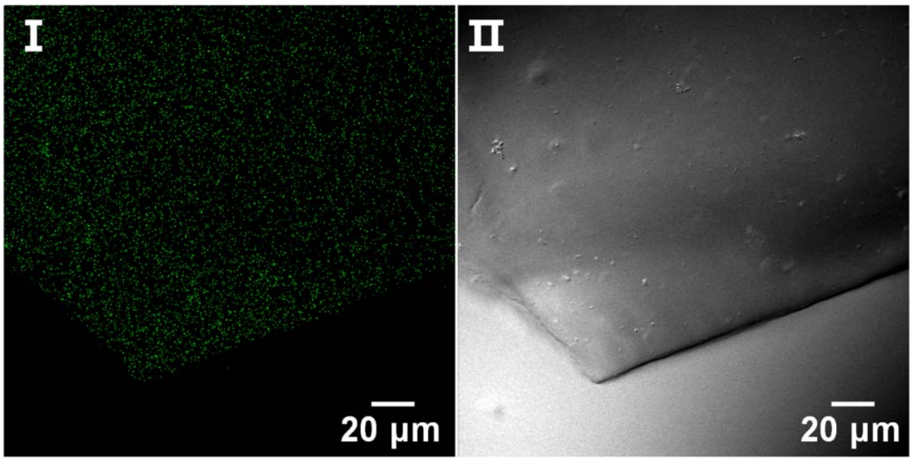

Supplementary Fig. 27 | Imaging of the doxorubicin-loaded hydrogel X. I, Confocal fluorescence microscopy and **II,** bright-field images of the doxorubicin-loaded hydrogel X.

b) While the mechanism for the release of doxorubicin from the hydrogel is complex and involves geometrical constrains of the hydrogel, concentration of the loaded drug, stiffness of the hydrogel and more. Nonetheless, we support the idea that the surface edges of the hydrogel play an important role in the release pattern of the drug. Supplementary Fig. 28 shows that a triangle-shaped hydrogel X in the absence of any triggers reaches an almost saturation value after two hours, and the further very slow release of the drug, presumably originates from its very slow diffusion to the hydrogel/solution boundary. Cutting this hydrogel into two equal pieces enhanced clearly the additional release of the drug. Presumably, the exposure of the new edges, generated by the cutting process, facilitates the additional release of the drug.

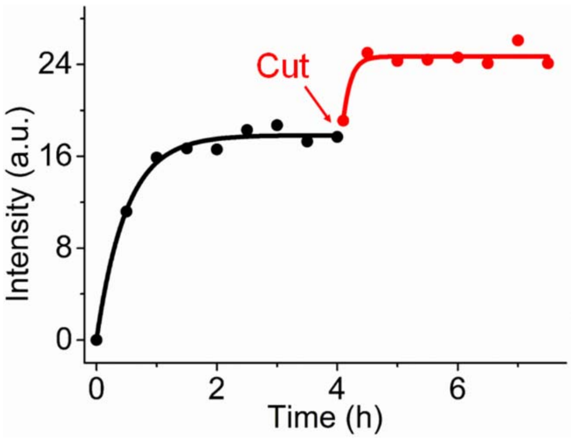

Supplementary Fig. 28 | Release of doxorubicin from CDN hydrogel X after cutting into two equal pieces. Time-dependent release profile of doxorubicin from the hydrogel X matrix in the absence of any triggers. After 4 hours, the matrix was cut into two equal pieces.
